# Variability in docking success rates due to dataset preparation

**DOI:** 10.1007/s10822-012-9570-1

**Published:** 2012-05-08

**Authors:** Christopher R. Corbeil, Christopher I. Williams, Paul Labute

**Affiliations:** Chemical Computing Group, Suite 910, 1010 Sherbrooke Street West, Montreal, QC H3A 2R7 Canada

**Keywords:** Docking, Scoring, Errors, MOE, GBVI/WSA

## Abstract

The results of cognate docking with the prepared Astex dataset provided by the organizers of the “Docking and Scoring: A Review of Docking Programs” session at the 241st ACS national meeting are presented. The MOE software with the newly developed GBVI/WSA dG scoring function is used throughout the study. For 80 % of the Astex targets, the MOE docker produces a top-scoring pose within 2 Å of the X-ray structure. For 91 % of the targets a pose within 2 Å of the X-ray structure is produced in the top 30 poses. Docking failures, defined as cases where the top scoring pose is greater than 2 Å from the experimental structure, are shown to be largely due to the absence of bound waters in the source dataset, highlighting the need to include these and other crucial information in future standardized sets. Docking success is shown to depend heavily on data preparation. A “dataset preparation” error of 0.5 kcal/mol is shown to cause fluctuations of over 20 % in docking success rates.

## Introduction

Docking methods have now been in existence for over 35 years, starting with Levinthal et al*.*’s use of docking to predict possible conformations of hemoglobin fibers [1]*.* Since then many docking programs have been developed [2], primarily for protein–ligand docking in the context of small-molecule structure-based drug discovery. While docking programs have become widespread, many issues remain unresolved such as the proper treatment of protein flexibility, solvation and ultimately, the accurate prediction of binding affinities [3–6]. To monitor improvements and the current status of the field, it has become popular to compare various docking methods with studies aimed at assessing the accuracy and limitations of the different programs and protocols. [7–10]. However, despite a large number of comparative studies, it still remains difficult to determine which programs and protocols result in overall performance improvements. Many studies have shown that docking success rates are heavily dependent on many variables, ranging from the scoring function being used [7], the target being investigated [8], the input for docking [9, 10], and even the metrics used to determine success in the study [11]. As a result, it can be a challenge to compare results from different validation studies, which often present contradictory conclusions.

One major stumbling block to the advancement of protein–ligand docking validation has been the lack of a standard test set agreed upon and used by the entire community. The absence of such a set is one reason why it can be difficult to compare or even reproduce published docking results, because access to the primary data used in the computational experiments is often limited [12]. The need for validation test sets has been partially addressed in other computational chemistry fields through competitions such as CSAR (Community Structure–Activity Resource) for binding affinity prediction [13, 14], CASP (Critical Assessment of protein Structure Prediction) [15–23] for protein structure prediction and CAPRI (Critical Assessment of PRediction of Interactions) [24–27] for protein–protein docking. In these events the data is curated by the organization and given to the participants (blinded or not) who then asses how their methods perform. The use of standard community tests sets in these competitions makes direct comparison of validation studies straightforward.

To overcome the lack of organized competitions and standardized test sets in protein–ligand docking, efforts have been made to publish datasets, such as Astex [28], and DUD [29], for use when conducting docking and/or binding affinity prediction experiments. However, even when docking studies use these published sets, it can be difficult to compare or reproduce results, because the researchers often significantly process and manipulate the data before using them as input for docking programs. The details of these manipulations are often subtle, and can have a profound effect on results; a small change in a hydroxyl rotamer in the binding pocket, or the inclusion or deletion of a bound water, can have huge effects on docking performance [30]. Unfortunately, exclusion of any of these small details from a methods section can make it difficult or impossible to reproduce published results.

With no standing organization to produce standardized datasets and to run competitions for protein–ligand docking, it is left up to individuals to organize fair and un-biased events [8, 13, 31–33]. One such event occurred in the “Docking and Scoring: A Review of Docking Programs” session during the 241st ACS national meeting in which we participated. The ultimate goal of this event was to assess the current status of docking programs. The session consisted of using the Astex Diverse Set [28] for pose prediction and the DUD set [29] for virtual screening accuracy. In a standardization effort the organizers of the competition prepared the input data themselves, and asked the participants to use the structures “as-given”. This would hopefully remove biases associated with dataset preparation and evolve some standardized datasets. The organizers also defined how the results should be reported, to minimize difficulties in comparisons that arise from using different success metrics.

This paper presents the results of our participation in this session and covers four major points of discussion:

1. Development of a new scoring function, GBVI/WSA dG.
2. The results of cognate docking with MOE using the ACS-Astex set input ‘as-given’ and after in-house manipulations.
3. Detailed analysis of docking failure cases, which point out errors and inconsistencies in source test datasets.
4. The effect of dataset preparation on docking error, and the effect of error on docking success rates.

The MOE docking architecture provides a standardized docking workflow that divides docking into a series of protocols, each of which can be modified and adjusted independently of the others. Thus it provides a good starting platform to compare the effect of each aspect of the docking workflow on the final results. To this effect, we developed a new scoring function, which was easily plugged into the existing MOE architecture for this study. We elected to develop a new scoring function instead of using existing functions because (a) we wanted to test a scoring function developed and trained on data *not* in the ACS-Astex set, (thus removing the bias of a scoring function trained to recover the Astex crystallographic pose) and (b) we wanted a simple force field-based scoring function with fewer terms, which will have a smaller probability of being over-fitted compared to more complex scoring functions [34].

The results of cognate docking to the ACS-Astex set are presented, both using the data ‘as-given’, as suggested by the competition organizers and after in-house manipulations. The performance improvements along with details and justification of the in-house input manipulations will be discussed.

Special attention is given to the docking failures, especially in cases where failure should be expected because of problems with the source data, such as bad contacts and incorrect chirality. We show that even the highly-curated ACS-Astex dataset used in this study has several problems, despite being of modest size and having been examined by experts in the field. This highlights the real technical and scientific difficulties in preparing protein–ligand validation test sets. Based on experiences in this study, recommendations for dataset preparation are put forth.

Finally, the estimated errors in docking that result from differences in structure preparation are shown to significantly affects docking, giving rise to differences in success rates greater than 20 %.

## Materials and methods

### Development of a force-field based scoring function

#### Dataset preparation

For training and testing of the GBVI/WSA dG (Generalized-Born Volume Integral/Weighted Surface area) scoring function, the SIE [35] and CSAR-NRC HiQ [13, 30] datasets where used respectively. The SIE set is comprised of 99 curated protein–ligand complexes with affinities spanning 10 orders of magnitude (−2 to −15 kcal/mol). The CSAR set is comprised of 343 high-quality, curated protein–ligand complexes with affinities spanning 12 orders of magnitude (1 to −17 kcal/mol).

Each set was downloaded from their respective sources [13, 35]. The structures were then minimized using the MMFF94x force-field with reaction-field electrostatics (Din = 1, Dout = 80) using a flat bottom tether (10.0 kcal/mol, 0.25 Å) which was applied to all atoms. All refinements were done in MOE [36].

#### Scoring function expression and model

The protein–ligand binding free energy is calculated using a formalism similar to that of SIE.
$E_{Inter}^{Coul.}$ and $E_{Inter}^{vdW}$ represent the columbic and van der Waals contribution to binding respectively. These terms were calculated using the MMFF94x force field using an 8–10 Å cutoff distance, with a dielectric constant of 1 calculated using MOE. The electrostatic solvation contribution, $\Updelta G_{Bind}^{R}$, is the change in reaction field energy upon binding. It is calculated using a continuum dielectric model with an interior dielectric constant of 1 and an exterior dielectric of 80. Reaction field energies were calculated using GB/VI [37] which estimates the free energy of hydration as a classical electrostatic energy plus a cavitation energy using a volume integral London dispersion energy. The $\Updelta G_{Bind}^{npsol}$ term represents the change in non-polar solvation (van der Waals and cavitation cost) upon binding. The $\Updelta G_{Bind}^{npsol}$ can be approximated using a weighted solvent-accessible surface area ($\Updelta SA_{weighted}$), scaled with at proportionality factor, γ.

$$ \Updelta G_{Bind}^{npsol} = \gamma \Updelta SA_{weighted} $$

**Fig. 1:**
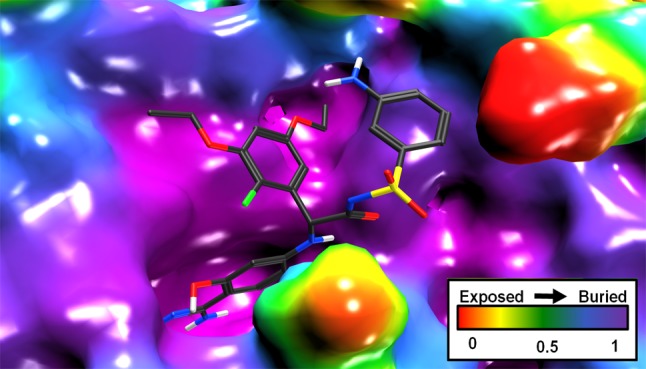
Solvent-accessible surface colored by pocket depth weight for 1YGC

Surface patches are weighted based on depth of the pocket, therefore down-weighting changes in exposed surfaced area (see Fig. 1).

α, γ and *c* are constants that were fit to the affinity values of the 99 complexes of the SIE dataset. Fits were done using partial least squares regression in the MOE QSAR module. Additionally the scaling factor for electrostatic interaction was empirically set to 2/3 which yielded higher accuracy then the ideal theoretical value of 1/2 [38].

### Preparing the ACS-Astex dataset

#### The ‘as-given’ ACS-Astex dataset

The initial dataset was prepared by the organizers of the ACS session and given to participants to use “as-given” [39]. Despite the organizers’ instructions, close inspection of the supplied data indicated that additional preparation was required. The PDB IDs of all the problem complexes are listed in Table 1, divided into sections based on three common types of problem. In 39 cases, the stereo configuration in SD file atom blocks were inconsistant with the supplied 3D geometries, and had to be reset. In 19 cases it was necessary to add hydrogens to co-factors. In 27 cases hydrogens were missing from alternate location B and were therefore added. Furthermore, in some cases alternate locations with the highest occupancy were not chosen for the receptor; instead the first alternate location “A” was used. In total, 58 out of 85 complexes required some minimal preparation. Lastly, the organizers identified 3 sites for 1TZ8 where site 2 and 3 are due to crystal contacts and therefore were removed from our statistics. These minimal preparations were discussed with the organizers and were deemed to be within the spirit of using the complexes as-given. This set of minimally-prepared structures will be henceforth referred to as the “as-given” set.

**Table 1:** Complexes requiring additional preparation

Inconsistent stereo configuration			
1GKC	1L7F	1R55	1VCJ
1GM8	1M2Z	1R58	1W1P
1GPK	1MMV	1R9O	1W2G
1HP0	1OF1	1S19	1X8X
1HVY	1OF6	1S3V	1XM6
1HWI	1OYT	1SQ5	1YGC
1HWW	1P2Y	1SQN	1YQY
1K3U	1P62	1TT1	1YV3
1KE5	1Q1G	1UML	1YWR
1KZK	1R1H	1V0P	
Co-factors with incorrect number of hydrogens			
1G9V	1KZK	1Q1G	1W1P
1HWI	1M2Z	1Q4G	1W2G
1IA1	1MMV	1R9O	1XM6
1J3J	1OPK	1T9B	1XOQ
1JJE	1P62	1TZ8	
Alternate locations missing hydrogens			
1GM8	1OPK	1T9B	1XOZ
1HNN	1OQ5	1TZ8	1Y6B
1HP0	1Q4G	1UOU	1YV3
1IA1	1R1H	1VCJ	1YWR
1KZK	1S19	1W1P	1Z95
1L2S	1S3V	1X8X	2BR1
1N46	1T46	1XOQ	

#### The ‘modified’ ACS-Astex dataset

**Fig. 2:**
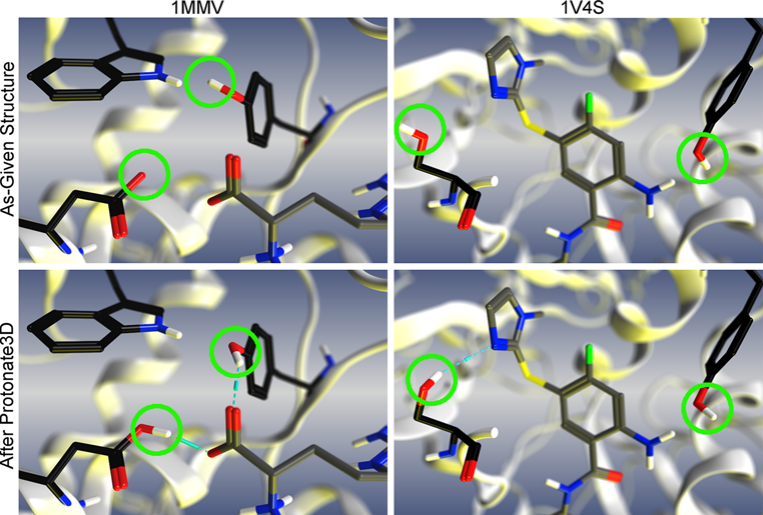
Examples of hydrogen bond network errors in ACS-Astex Set. The *As*-*Given* represent the initial structures given to participants by the organizers, while Protonate3D structures are the structure after using Protonate3D to re-optimize the hydrogen bond network

Upon closer examination of some of the complexes it was noted that the hydrogen bond network was not optimal and therefore further optimization was warranted. Two examples demonstrating the need for re-optimization are 1MMV and 1V4S (see As-Given Structures in Fig. 2). In 1MMV the given structure had the hydroxyl of Tyr562 oriented toward a Trp561 creating a clash. In addition either the carboxylate of the Asp597 or the ligand should be protonated to create a hydrogen bond. In the case of 1V4S the orientation of the hydroxyls of a Ser64 and Tyr215 where not positioned correctly and in one case caused clashing with the ligand.

To create an in-house ‘prepared’ version of the ACS-Astex dataset, the SEQRES records from the original PDB files were downloaded [40] and used to cap chain termini and chain breaks with ACE and NME groups. The PDB IDs of structures requiring capping are listed in Table 2. The hydrogen bond networks were re-optimized using Protonate3D [41], which optimizes hydroxyl and thiol rotamers, His/Asp/Glu tautomers, acid/base/metal ionization states, and Asn/Gln/His flips (see Protonate3D Structure in Fig. 2). The structures were then energy minimized in MOE to a gradient of (0.05 kcal/mol/Å) using the MMFF94x force-field with reaction-field electrostatics (Din = 1) and flat-bottom tethers (10.0 kcal/mol, 0.25 Å) applied to each atom. This minimization is quite constrained and results in an average heavy atom RMSD from the initial coordinates of 0.23 Å for protein atoms (both in the binding pocket and the entire receptor) and 0.18 Å for ligand atoms. This set of minimally-prepared structures will be henceforth referred to as the “modified” set.

**Table 2:** Complexes requiring capping of chain termini or chain breaks

Complexes requiring capping of chain termini			
1GKC	1MEH	1Q41	1V48
1GM8	1 MMV	1R55	1V4S
1GPK	1 MZC	1R9O	1W1P
1HNN	1N1M	1S19	1W2G
1HP0	1N2J	1SJ0	1XM6
1HWI	1N2V	1SQN	1XOQ
1HWW	1N46	1T46	1XOZ
1IG3	1OF1	1T9B	1Y6B
1J3J	1OF6	1TT1	1YGC
1JD0	1OPK	1TZ8	1YQY
1JJE	1OQ5	1U1C	1YV3
1JLA	1OYT	1U4D	1YVF
1K3U	1P2Y	1UML	1YWR
1LPZ	1P62	1UNL	2BM2
1LRH	1PMN	1UOU	2BR1
1M2Z	1Q1G	1V0P	2BSM
Complexes requiring capping of chain breaks			
1GPK	1N46	1SJ0	1V48
1HP0	1NAV	1SQ5	1W2G
1HWI	1OF1	1SQN	1XM6
1J3J	1OF6	1T46	1XOQ
1JLA	1OYT	1T9B	1Y6B
1KE5	1P62	1U1C	1YV3
1L2S	1PMN	1U4D	1YWR
1MEH	1Q41	1UOU	1Z95
1MMV	1R9O	1V0P	2BR1

#### The ‘corrected’ ACS-Astex dataset

After the session transpired at the ACS meeting, 3 structures from the ACS-Astex set (1GPK, 1HVY and 1S3V) were identified as containing an inverted stereocenter in the original dataset given to the participants compared to the original PDB structure. Thus, docking results that use the corrected version of these ligands (where the stereocenters were set to be the same stereochemistry as in the PDB) will be referred as the “corrected” set.

### Docking methodology

#### MOE docking architecture

The MOE-Dock architecture consists of four major components: (1) ligand-conformation generation (2) optional pharmacophore filtering (3) ligand placement and scoring in the pocket, and (4) flexible receptor and ligand refinement with re-scoring. In this study, ligand conformation generation was accomplished by supplying the docking engine with an ensemble of prepared ligand conformations generated using the Conformation Import application [36], with default parameters modified as follows to increase the number of conformations: filters were removed, fragment strain and total strain limits where set to 10 kcal/mol, and the maximum number of outputted conformations was set to 10,000. The resulting ensemble was then minimized using MMFF94x and partial charges were assigned to the atoms.

The binding site region was defined using the crystallographic ligand for all datasets. Since the purpose of the study was to demonstrate the upper limit of docking accuracy on the simple problem of self-docking, the default “Rigid Receptor” protocol was used [36], as opposed to flexible receptor/induce fit options. Ligand placement was performed using the Triangle Matcher protocol, which defines the active site using α-spheres [42] similar to both the α-spheres in the MOE-SiteFinder application and the spheres generated by DOCK [43]. Ligands are placed by superposing triplets of ligand atoms onto triplets of α-spheres, followed by removing poses which clash with the protein. The search is exhaustive for small molecules.

The top 1,000 poses produced from placement were then scored using the London dG scoring function [36].

$$ \Updelta G_{LdG} = c + E_{flex} + \sum\limits_{{h{ - }bonds}} {c_{hb} f_{hb} + \sum\limits_{{metal{ - }lig}} {c_{m} f_{m} + \sum\limits_{atoms i} {\Updelta D_{i} } } } $$

Here *c*, *c*_*hb*_ and *c*_*m*_ are constants which have been trained on over 400 protein ligand complexes. *E*_*flex*_ is a topological estimate of ligand entropy. Both *f*_*hb*_ and *f*_*m*_ are measures of geometric imperfections of protein–ligand and metal–ligand interactions. $\Updelta D_{i}$ is the desolvation energy term which is approximated using a volume integral London dispersion similar to that found in GB/VI [37]. The top 30 poses as ranked by London dG are kept and minimized using MMFF94x within a rigid receptor. The resulting poses are then scored using the new GBVI/WSA dG scoring function described previously.

Ligand placements were assessed with the root-mean-squared-deviation (RMSD) between the heavy atoms of the predicted pose and those of the crystal structure. The percent success (% success) for placement was defined as the number of systems where the RMSDs to the crystal structure of a docked pose is less than a given threshold.

## Results and discussion

### GBVI/WSA dG scoring function development

The SIE set was selected for training the scoring function because the SIE scoring function formalism is similar to that of the proposed GBVI/WSA dG scoring function. Additionally, the SIE scoring function has been shown to be predictive in various tests and applications [44–51], demonstrating the usefulness of the SIE functional form, and the use of its corresponding set for training of GBVI/WSA dG. The CSAR dataset was used to test the scoring function due to its increased size, range and number of protein families. Additionally the CSAR set has been applied to many scoring functions, enabling easy comparison, and has proven to be a challenging set [14].

Training of the GBVI/WSA dG scoring function on the SIE dataset resulted in a mean-unsigned error (MUE) of 1.35 kcal/mol which is significantly better than the null model, where the average affinity of the set is used as the predictor (MUE = −2.38 kcal/mol; see Table 3). The GBVI/WSA dG scoring function also performs slightly better than any of its components or combination thereof. The results for GBVI/WSA dG also compare well with those of SIE (MUE = 1.34 kcal/mol) for the training set [30].

**Table 3:** Results of various scoring function models on SIE training set

	MUE (kcal/mol)	RMSE (kcal/mol)	R^2^
GBVI/WSA dG	1.35	1.61	0.70
SIE	1.34	1.76	0.65
NULL	2.38	2.96	0.00
vdW	1.89	2.33	0.38
Ele	2.37	2.96	0.00
WSA	1.57	1.88	0.59
vdW + Ele	1.43	1.74	0.65
vdW + WSA	1.49	1.82	0.62
Ele + WSA	1.58	1.88	0.60

**Fig. 3:**
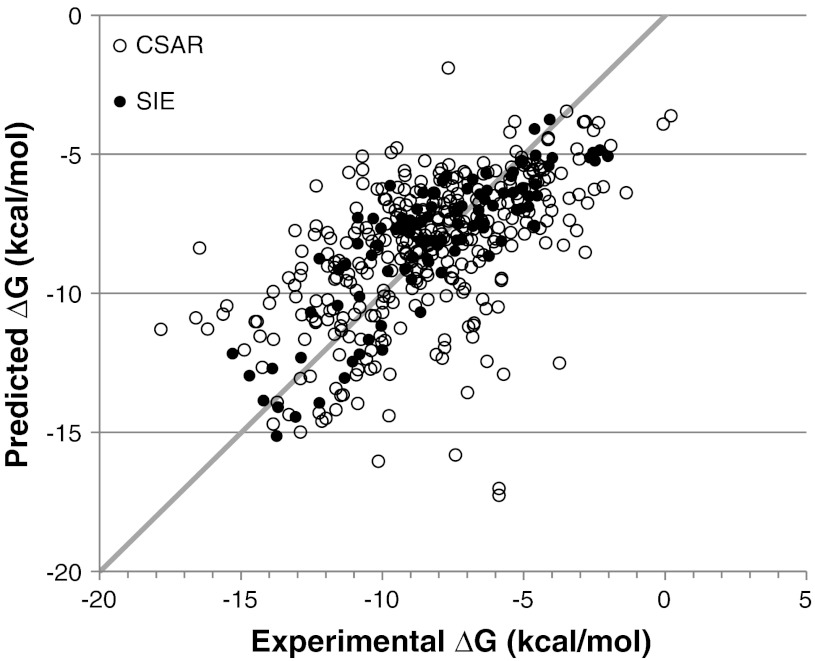
Predicted vs experimental binding affinities for GBVI/WSA dG scoring function for SIE (training) and CSAR (testing) sets. *Gray line* represents ideality

Application of the GBVI/WSA dG scoring function to the CSAR-NRC HiQ (see Table 4, Fig. 3) set gave rise to a MUE of 2.09 kcal/mol which are slightly better than the null model (MUE = −2.42 kcal/mol) and mirror SIE’s results on the same dataset (MUE = 1.98 kcal/mol). The slight degradation in performance when moving from training to testing was deemed acceptable, and GBVI/WSA dG was used for the remainder of the study. Although the MUE is significant when compared to binding energies, it should be noted that accurate continuum solvation energies can only achieve MUEs in the range of 1.5–1.8 kcal/mol [33]; therefore one should not expect the accuracy of a scoring function such as GBVI/WSA dG to be greater than one of its components.

**Table 4:** Results of various scoring function models on CSAR-NRC HiQ set

	MUE (kcal/mol)	RMSE (kcal/mol)	R^2^
GBVI/WSA dG	2.09	2.73	0.30
SIE	1.98	2.49	0.38
NULL	2.42	3.04	0.00

### Cognate docking with ACS-Astex sets

**Fig. 4:**
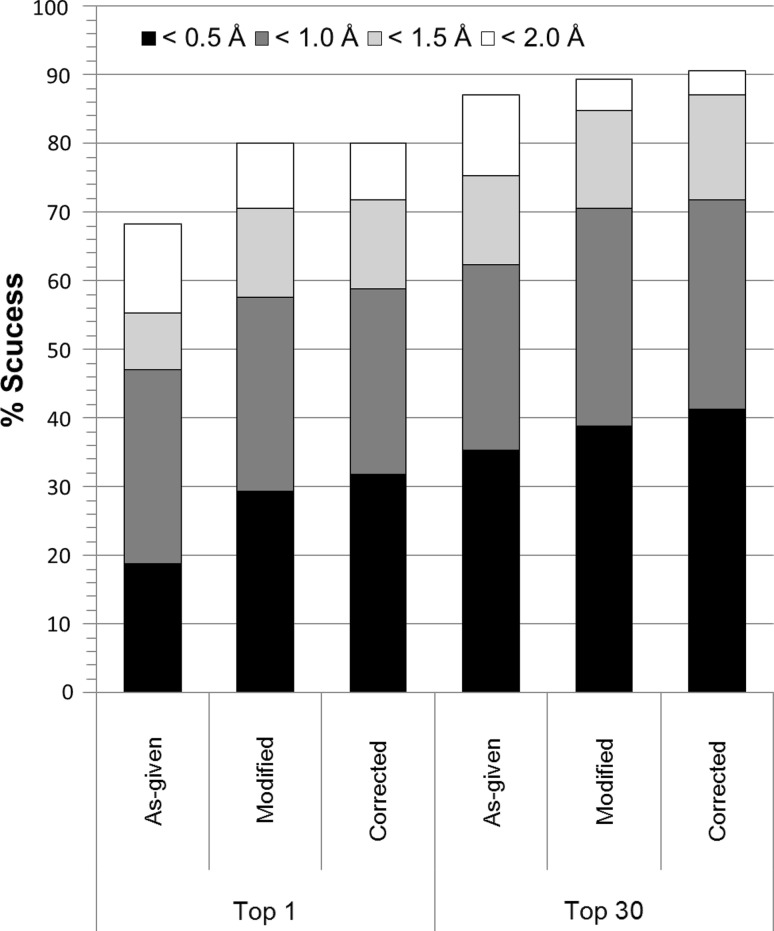
Docking success rates for MOE on as-given, modified and corrected ligands ACS-Astex sets for best scoring and lowest RMSD in top 30

The cognate docking experiments were performed with MOE and the newly developed GBVI/WSA dG scoring function using the “Rigid Receptor” protocol. The input data-sets described in the “Methods” section were used; the ACS-Astex ‘as-given’, ACS-Astex ‘modified’ and ACS-Astex ‘corrected’ sets. The results of docking with the three data-sets are reported in Fig. 4 as the % success for the ‘top 1’ and ‘top 30’ poses at four different RMSD thresholds −0.5, 1.0, 1.5 and 2.0 Å. The % success for the ‘top 1’ is the percentage of systems where the top-scoring docked pose has an RMSD to the crystal pose less than the RMSD cut-off, while % success for the ‘top 30’ is the percentage of systems where *any* pose in the top 30 docked poses has an RMSD to the crystal structure less that the cut-off.

At the 2 Å RMSD threshold docking with the ACS-Astex ‘as-given’ dataset resulted in 68 % success for the top 1 and 87 % for the top 30 poses. The 19 % difference in percent success between the top 1 and top 30 poses are indicative that we have more scoring failures than placement failures. Since force fields are sensitive to system preparation this result initially motivated the creation of the modified and corrected versions of the ACS-Astex set.

Docking with the ACS-Astex ‘modified’ dataset resulted in increased % success for the top 1 and top 30 poses at all RMSD thresholds. At the 2 Å threshold the % success is 80 and 91 % respectively for the top 1 and top 30 poses. After the ACS-session the organizers discovered that 3 of the 85 structures (1GPK, 1HVY and 1S3V) had inverted ligand stereochemistry when compared to the PDB structures. These inversions were present in the input data given to us by the organizers [39]. Incorporating these corrections and re-docking improved the % success rates even further. After re-docking with the corrected ligand stereochemistry 1GPK and 1S3V successfully docked with better scores and RMSDs (RMSD of top 1: 1GPK = 0.31 Å, 1S3V = 0.39 Å), while 1HVY remained unimproved for the top 1 pose. However, docking the corrected 1HVY ligand did produce a pose under 2 Å RMSD in the top 30 (RMSD of top 30 = 1.39 Å), and thus improved the top 30 result. Overall, docking with the ‘modified’ ACS-Astex data improved results over the ‘as-given’ data, and the stereochemical corrections improved results further still (docking success rates: top 1 = 80 %, top 30 = 91 %). Furthermore, the difference in the % success between the top 1 and top 30 poses is smaller for the corrected set (11 % for correct set vs 19 % as-given), suggesting scoring has improved by using the corrected versus the as-given set.

The improvement in docking results going from the ‘as-given’ to the ‘modified’ and ‘corrected’ datasets is also reflected in the pose RMSD statistics give in Table 5, which reports the mean, median, standard deviation, minimum and maximum of pose RMSDs across three data-sets. For the ‘as-given’ dataset the median RMSD for top 1 and top 30 poses are 1.21 (mean = 2.05 Å) and 0.73 Å (mean = 1.06 Å) respectively. The median RMSDs improve when the ‘modified’ and ‘corrected’ dataset are used for docking, with top 1 pose median RMSDs dropping to 0.88 and 0.87 Å respectively for the modified and corrected sets, and top 30 pose median RMSDs dropping to 0.67 and 0.64 Å.

**Table 5:** Statistical performance of MOE on As-given, Modified and Corrected ACS-Astex sets for best scoring and lowest RMSD in top 30

	Top 1			Top 30		
	As-given	Modified	Corrected	As-given	Modified	Corrected
Mean	2.05	1.30	1.27	1.06	0.91	0.88
SD	2.06	1.16	1.15	0.94	0.74	0.72
Median	1.21	0.88	0.87	0.73	0.67	0.64
Min	0.16	0.13	0.13	0.16	0.13	0.13
Max	8.58	4.96	4.96	5.10	3.04	3.04

The decrease in median RMSD for the top 1 pose between the ‘as-given’ and ‘corrected’ data-sets reflects improvements in scoring achieved by using the ‘corrected’ versus the ‘as-given’ data-set. This increase in docking accuracy by using optimized structures as input has been seen with other docking programs [7] which recommend optimizing the protein structure in the presence of its cognate ligand prior to docking [52–55]. Even though heavy atom refinement prior to docking biases the binding pocket to its cognate ligand, in our case the changes upon refinement are small (less than 0.23 Å RMSD for the pocket) and well within the resolution of the crystal structures used. Since the purpose of this session was to assess the highest possible level of accuracy in self-docking, the refinement protocol seemed acceptable, especially because it is common practice in other self-docking protocols. Additionally a previous study [7] has shown that while refinements can improve accuracy in cognate docking, they do not affect cross-docking accuracy, suggesting that relaxing the protein biases self-docking results, but not cross-docking results.

### Detailed analysis of docking failures

#### Known structural problems with the Astex set

For purposes of this study a docking failure is defined as cases where the top scoring pose has an RMSD to the crystal structure of greater than 2 Å. Based on this criterion, 17 out of 85 ACS-Astex complexes (20 %) would be considered failures. The PDB codes and details of the failure are reported in Table 6. Failure cases will be examined in detail to highlight the types of problems we encountered.

**Table 6:** Docking failures on corrected ACS-Astex set

PDB code	Failure type	Minimum RMSD (Å)		Present in binding site	
		Top 1	Top 30	Water	Metal
1g9v	Placement	2.51	2.24	X	
1gm8	Placement	3.20	2.69	X	
1hp0	Scoring	2.93	1.07		X
1hvy	Scoring	2.13	1.39	X	
1jd0	Scoring	4.96	1.68		X
1l2s	Scoring	3.65	0.80		
1mzc	Placement	3.66	2.92		X
1n2v	Scoring	2.23	1.14		
1oq5	Scoring	3.47	1.00		X
1owe	Scoring	3.18	1.09	X	
1q1 g	Scoring	2.28	1.91	X	
1r58	Placement	2.86	2.84		
1sq5	Placement	4.96	2.55	X	
1xm6	Scoring	2.40	0.50	X	X
1y6b	Placement	4.72	2.97		
1ygc	Placement	3.04	3.04	X	

Nearly half of the failure cases (8) were due to placement failure, where the docker was unable to generate any pose under 2 Å RMSD. In six other cases, failure was due to scoring, because a pose was generated under 2 Å but it was not scored as the top pose.

The organizers identified many structural errors present in 22 complexes which normally exclude them being selected as part of a docking set. These problems include complexes which contain poor electron density of the ligand and/or binding pocket residues, alternate location of residues close to binding pocket and possible crystal packing interactions with the ligand. Surprisingly only 2 of the 22 problem structures docked unsuccessfully and made it to the failure list. This included 1HVY which was identified as having crystal packing interactions with the ligand and 1Y6B which was identified as having an alternate location of a residue in the binding site. To see if 1HVY failed due to exclusion of the crystal packing interactions, symmetry-related residues with at least one atom within 10 Å of any atom in the asymmetric unit were created. This new structure was then used for re-docking of the 1HVY ligand. In the case of 1Y6B, it was re-docked using the lower occupancy conformation of CYS1043. In both cases re-docking did not yield a successful result. This suggests that the identified structural problems were not causing the failures to produce successful docking results.

#### Case studies of docking failures

Further examination of the 17 failures suggested that many could be attributed to a lack of bridging water molecules, metal binding interactions and predictions in solvent-exposed regions of ligands.

Of the 17 complexes which failed to dock successfully, 8 fail due to the lack of bridging water molecules (these are noted in Table 6). Failure due to bridging water effects is a well-known issue in the field of docking [2, 3, 56, 57], which is addressed in some docking programs by allowing displacement of water or by treating water as part of the receptor [58–63]. However, for this study they were deleted by the organizers and could not be re-added by the participants.

**Fig. 5:**
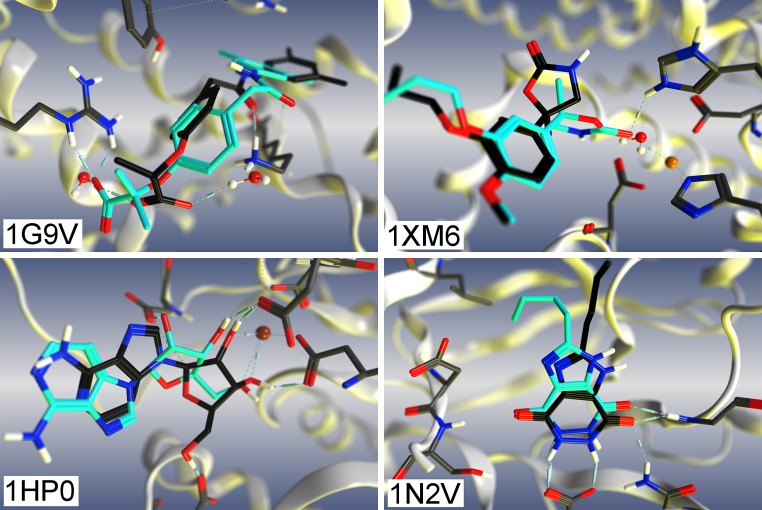
Examples of various self-docking failures on ACS-Astex modified set. *Charcoal* crystal structure pose, *Cyan* docked pose

One example of a crucial bridging water in the ACS-Astex set is PDB code 1G9V (shown in Fig. 5), where the crystallographic pose of the ligand (green) clearly interacts through 2 bridging water molecules with a lysine and an arginine. Because the waters are not present in the ACS-Astex set, the docking engine cannot generate a single pose under 2.0 Å RMSD (Top 30 = 2.24 Å RMSD) and therefore favors interacting directly with the arginine (Top 1 = 2.51 Å RMSD).

Another example of a crucial water molecule is in PDB code 1XM6, where a water molecule tightly bound to a metal blocks ligand access to the metal. The ligand does not coordinate to the zinc in the crystal structure, due to difficulty associated with displacing the water upon ligand binding. With the water absent, a pose that resembles the crystal structure is generated (Top 30 = 0.5 Å RMSD) but it scores 0.5 kcal/mol higher in energy than the best scoring pose (Top 1 = 2.40 Å RMSD) which binds to the zinc. The exclusion of the crystallographic water from this complex is the root of this docking failure even though it is not bridging between the ligand and receptor. To accurately model this system one should not only include the water but be able to accurately model the displacement of the water and the associated displacement cost.

Predicting metal interactions is a recognized issue in the field of docking and scoring [2]. While there are methods which identify free coordination sites on a metal [7, 64, 65], identifying how or if the ligand binds remains difficult. This is exemplified with 1HP0 where in the crystal structure, the ligand coordinates through the O2’ and O3’ of the sugar, while the best scoring pose coordinates through O3’ and O5’. In fact the lowest RMSD pose found (Top 30 = 1.03 Å RMSD) is only 0.02 kcal/mol higher in energy then the best scoring pose (Top 1 = 2.93 Å RMSD).

Another common docking failure is the scoring of solvent exposed regions of the ligand such as with 1N2V. In the crystal structure the butyl group of the ligand is solvent exposed making no strong interactions with the protein. While we can generated a good pose (Top 30 = 1.14 Å RMSD) it is over 0.5 kcal/mol higher in energy than the best scoring pose (Top 1 = 2.23 Å RMSD). The docked pose is favoured because the butyl group is placed into a pocket which is filled with water in the crystal structure.

Overall, many of the failures cases can be attributed either missing information in the curated dataset or the accuracy of the scoring function. Although the competition and the dataset produced are steps in the right direction, the results here suggest that future curated docking datasets need to contain all the information from the crystal structure. The results also demonstrate the necessity of including crystallographic waters to predict the correct pose in some cases. While the addition of crystallographic waters have been shown to increase docking accuracy, it must be noted that there is no improvement when including them for binding affinity predictions [34].

### Effect of dataset preparation errors on docking success rates

Even though a respectable success rate was achieved on ACS-Astex set, this was only achieved by preforming additional preparation to the structures that were initially given. This additional preparation accounted for an increase in 11 % accuracy when compared to running on the ‘as-given’ set. This suggests that the GBVI/WSA dG scoring function is sensitive to minor modifications in the protein environment which is typical of any force-field based scoring function. This sensitivity can have a dramatic effect on the accuracy of docking and binding affinity predictions. As an example, Sulea et al*.* found that correcting multiple structural problems in the CSAR set resulted in decreasing the MUE by 0.5 kcal/mol for SIE [30].

**Fig. 6:**
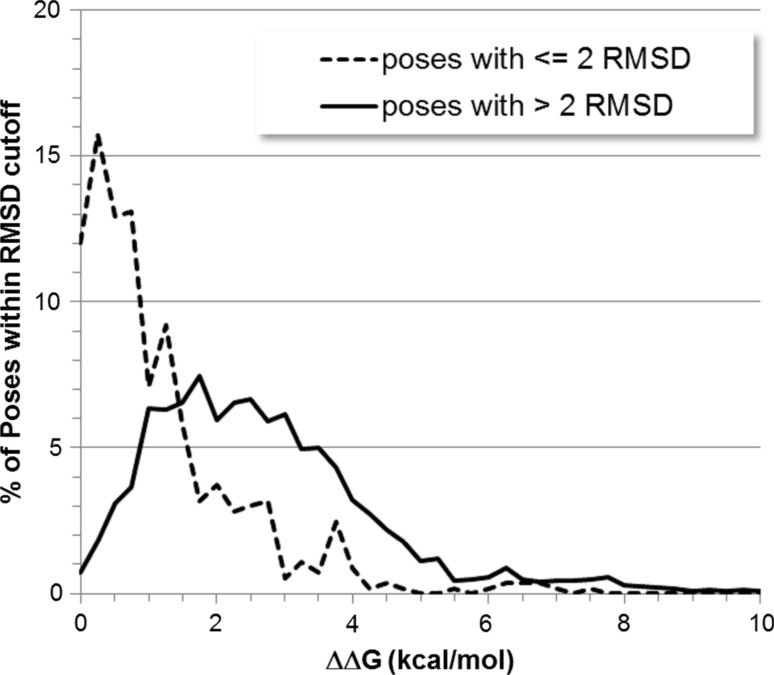
Plot of the normalized percentage of poses at various ΔΔG values. Poses with RMSD less than 2 Å RMSDs are plotted with a *solid line* and poses with RMSDs greater than 2 Å RMSD are plotted with a *dashed line*

To accurately distinguish between active and decoy ligands in virtual screening, there must be good separation in predicted binding affinities between actives and decoys. In the case of pose prediction one needs significant separation in energy between good (≤2 Å RMSD) and bad (>2 Å RMSD) poses. In other words the further the pose is from the crystal structure the higher in energy it should be. On average, in reference to docking using MOE on the ACS-Astex set, good poses are within 1.07 kcal/mol of best scoring pose, while bad poses are 2.74 kcal/mol. This difference of 1.67 kcal/mol may seem large when compared to the 0.5 kcal/mol error associated with dataset preparation but is close to the limit of accuracy of the GBVI/WSA dG scoring function (MUE on training set = 1.4 and 2.1 kcal/mol on testing set). In fact by examining the number of docking poses versus their relative energy difference with the best scoring pose, the good and bad poses overlap significantly (see Fig. 6). This suggests that even if we can always generate a good pose (no placement failures), we will not always be able to identify it (scoring failure) since many poses are similar in energy and competing with the best scoring pose.

To assess the error associated with re-ranking of competing poses, an error perturbation analysis was performed. The analysis was done by introducing a uniform distributed random error of ± 0.25 kcal/mol to all poses and repeated for 10 000 iterations. The 0.5 kcal/mol energy window was selected to approximate the error associated with dataset preparation and how this can affect our docking success rate. After the introduction of the error the docked poses were re-ranked. The best scoring pose was then identified and used to generate the statistics for each complex (see Table 7) and the dataset as a whole.

**Table 7:** Statistical performance of MOE on corrected ACS-Astex set for single point and docking results after error pertubation of ±0.25 kcal/mol

	RMSD (Å)	
	Single point	Error simulation
Mean	1.27	1.35
SD	1.15	1.15
Median	0.87	0.95
Min	0.13	0.24
Max	4.96	4.80

**Fig. 7:**
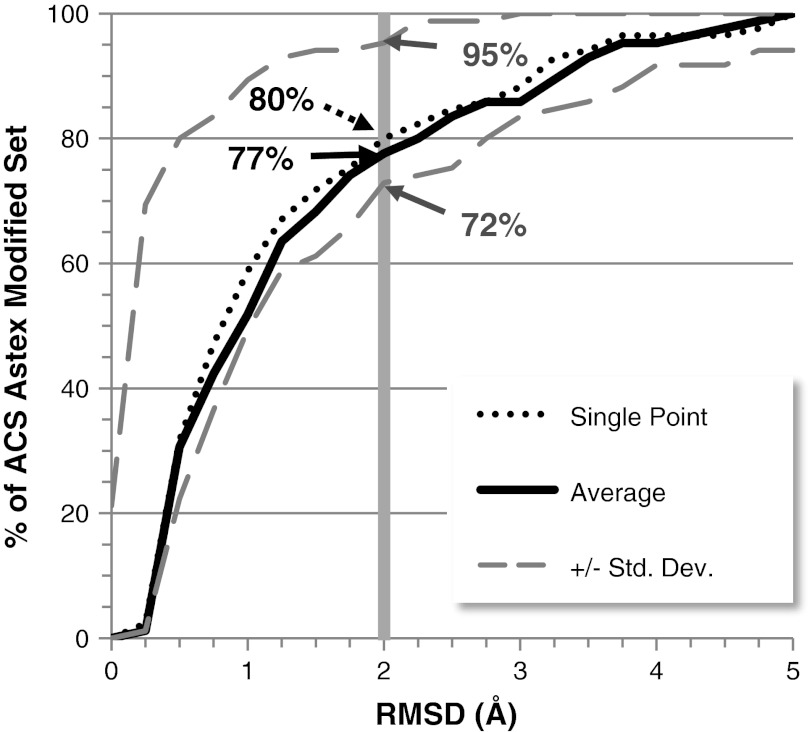
Success rate for ACS-Astex correct set versus RMSD after error simulation of ±0.25 kcal/mol. The *dotted line* represents the success rate prior to the simulation, *solid black* for the average, dashed lines for +/− standard deviation.

Overall performance is degraded when accounting for the possibility of re-ranking due to error (which is expected). The error perturbation allows estimation of the effect of data preparation or force field error on docking success rate. Within one standard deviation of the average RMSD for a complex, the success rate at 2 Å RMSD can vary from 72 to 95 % (see Fig. 7). Of note is that the lower bound is similar to the docking success rate when using the ACS-Astex ‘as-given’ set. The error perturbation demonstrates that minor changes in dataset preparation and the scoring differences that result can have a dramatic effect of the success rate of docking. It also suggests why it may be difficult to accurately compare virtual screening recall rates from docking programs, since there are no standard protocols for dataset preparation in docking, and differences in data preparation can result in scoring differences that can significantly affect recall rates.

## Conclusion

The MOE docking engine with the newly developed GBVI/WSA dG scoring function was found to produce a top-scoring pose within 2 Å of the X-ray structure for 80 % of the Astex targets. For 91 % of the targets, a docked pose less than 2 Å from the X-ray structure was produced within the top 30 poses. Docking performance increased significantly when reasonable modifications to the source data, such as re-optimizing the hydrogen bond network, capping chain breaks and termini, relieving steric clashes, and other minor changes, were applied. Many cases of docking failures were found into be caused by the absence of bound waters in the source data, suggesting waters and other bound species should be included in future standardized sets.

Despite great efforts by experts in the field to prepare the data for this competition, significant problems with the data were still found, highlighting how difficult (and painful) it can be to compile, curate and maintain a docking test set. The number of errors and the details surrounding each error case suggest that the probability of a single person flawlessly preparing and maintaining an entire docking dataset is low, especially as the dataset becomes large. Other datasets used as docking standards are also known to contain errors [29, 66, 67], yet these datasets continue to be used without being corrected. Thus, the difficulty in preparing standard docking datasets, coupled with the heavy dependence of docking results on data preparation, suggests that a “data preparation error” should *always* be include in docking validation studies, at least to indicate the upper and lower bounds of performance. This work shows that dataset preparation errors as small as 0.5 kcal/mol can cause fluctuations of over 20 % in docking success rates.

The results of this study suggest a series of recommendations for future docking dataset preparation:

1. Future datasets should consist of only *adding* additional data, such as correcting chain termini, capping chain breaks and adding missing side chains. Information such as alternate conformations of residues and crystallographic waters should also be retained. Any decision to *remove* information from the structure should be decided by the researcher, after retrieving the curated set.
2. The curated dataset must be updated when an error is identified and not allowed to propagate. If the problem is with the experimental data and cannot be updated, the structure should be removed from the set.
3. Future datasets should move beyond self-docking (which was done in this study) since proteins are dynamic objects and therefore is a best case scenario only and include multiple structures of the same protein with different ligands (cross-docking).
4. Benchmarking sets should be prepared, curated and stored by the community as a whole, such as the multiple revisions to the CSAR set. Until a common community built dataset is created, it will be difficult to draw conclusions from a comparative docking study.
5. Until a universal docking data preparation protocol is developed and accepted by the community, docking studies should always consider the effect of dataset preparation on docking performance, including estimates of the magnitude of dataset preparation error, and its effect on the reported docking performance.

When a docking dataset created with these recommendations is available we can better assess docking methods and move towards creating blinded competitions such as CSAR, CASP and CAPRI. Through competitions like these the field of docking will hopefully become more robust and reliable.
